# Health systems resilience in practice: a scoping review to identify strategies for building resilience

**DOI:** 10.1186/s12913-022-08544-8

**Published:** 2022-09-19

**Authors:** Lena Forsgren, Fabrizio Tediosi, Karl Blanchet, Dell D Saulnier

**Affiliations:** 1 Department of Global Public Health, Karolinska Institutet, Tomtebodavägen 18a, 171 77 Stockholm, Sweden; 2 Swiss Tropical and Public Health Institute, Kreuzstrasse 2, 4123 Allschwil, Switzerland; 3 University of Basel, Petersplatz 1, P. O. Box, 4001 Basel, Switzerland; 4 Geneva Centre of Humanitarian Studies, Faculty of Medicine, University of Geneva, 28, Boulevard du Pont-d’Arve, 1205 Geneva, Switzerland; 5 Department of Clinical Sciences Malmö, Lund University, Jan Waldenströms gata 35, 214 28 Malmö, Sweden

**Keywords:** Health systems, Resilience, Scoping review

## Abstract

**Background:**

Research on health systems resilience has focused primarily on the theoretical development of the concept and its dimensions. There is an identified knowledge gap in the research on how to build resilience in health systems in practice and ‘what works’ in different contexts. The aim of this study is to identify practical strategies for building resilient health systems from the empirical research on health systems resilience.

**Methods:**

A scoping review included empirical research on health systems resilience from peer-reviewed literature. The search in the electronic databases PubMed, Web of Science, Global Health was conducted during January to March 2021 for articles published in English between 2013 to February 2021. A total of 1771 articles were screened, and data was extracted from 22 articles. The articles included empirical, applied research on strategies for resilience, that observed or measured resilience during shocks or chronic stress through collection of primary data or analysis of secondary data, or if they were a review study of empirical research. A narrative summary was done by identifying action-oriented strategies, comparing them, and presenting them by main thematic areas.

**Results:**

The results demonstrate examples of strategies used or recommended within nine identified thematic areas; use of community resources, governance and financing, leadership, surveillance, human resources, communication and collaboration, preparedness, organizational capacity and learning and finally health system strengthening.

**Conclusions:**

The findings emphasize the importance of improved governance and financing, empowered middle-level leadership, improved surveillance systems and strengthened human resources. A re-emphasized focus on health systems strengthening with better mainstreaming of health security and international health regulations are demonstrated in the results as a crucial strategy for building resilience. A lack of strategies for recovery and lessons learnt from crises are identified as gaps for resilience in future.

**Supplementary Information:**

The online version contains supplementary material available at 10.1186/s12913-022-08544-8.

## Background

Interest in health systems resilience has been growing amongst policy makers and researchers over the last fifteen years and has grown in popularity following major crises like the 2008 financial crisis, the outbreak of the Ebola virus in West Africa during 2014 to 2016, and the ongoing COVID-19 pandemic. Health systems resilience has been defined *“as the systemic attribute of persistence in the face of chronic stress and acute shocks, and as one that not only allows health systems to absorb but has the potential to support them to adapt and transform when faced with shorter or long term challenges”* [1]*.* Although health systems resilience is a relatively new concept in the health sector, several definitions and theoretical health system resilience frameworks have been developed [2, 3]. However, it has not yet been extensively operationalized in health system research. How theory is translated into evidence and then implementation research is crucial to define what strategies may work under which circumstances to be able to build the resilience of health systems [2].

To date, there is little knowledge on what strategies have been used and seen as successful to strengthen health systems resilience in different contexts. Identifying such strategies from the literature could provide guidance on future actions to implement before, during, or after a crisis to health system managers, policy makers, or decision makers [4]. The aim of this scoping review was to identify and describe effective strategies on health systems resilience that are documented in the literature on empirical health systems resilience research.

## Methods

A scoping review [5] was conducted during January to March 2021. A scoping review was chosen because of the expected heterogeneity of resilience strategies across the body of research evidence. The review included research that defined itself as explicitly investigating the resilience of health systems, via the aims and objectives, study design, or methods (Table 1). Studies were included if they were empirical, applied research that observed or measured resilience during shocks or chronic stress through collection of primary data or analysis of secondary data, or if they were a review study of empirical research. Articles needed to be peer-reviewed and published in a scientific journal in English between January 2013 and February 2021. The search years were chosen based on an earlier search which found that the majority of empirical research was published after 2012 [2]. Papers were excluded if they focused micro or macro level health service delivery without linking the findings to the meso level of the health system or the system’s other functions.

**Table 1:** Eligibility criteria for the studies

**Inclusion criteria**
Full text written in English
Published between 2013 and 2021
Empirical research and evidence synthesis papers
Peer-reviewed articles published in a scientific journal
Articles that operationalize resilience by using resilience concepts in study designs and methods
**Exclusion criteria**
Individual/psychological resilience (including resilience of health care providers)
Resilience that focuses on delivery of health services, like patient safety, without link to the broader health system and its functions
Resilience that is not related to health systems (e.g. development, agriculture, climate)
Editorials, commentaries, opinion papers, and conference abstracts

**Fig. 1:**
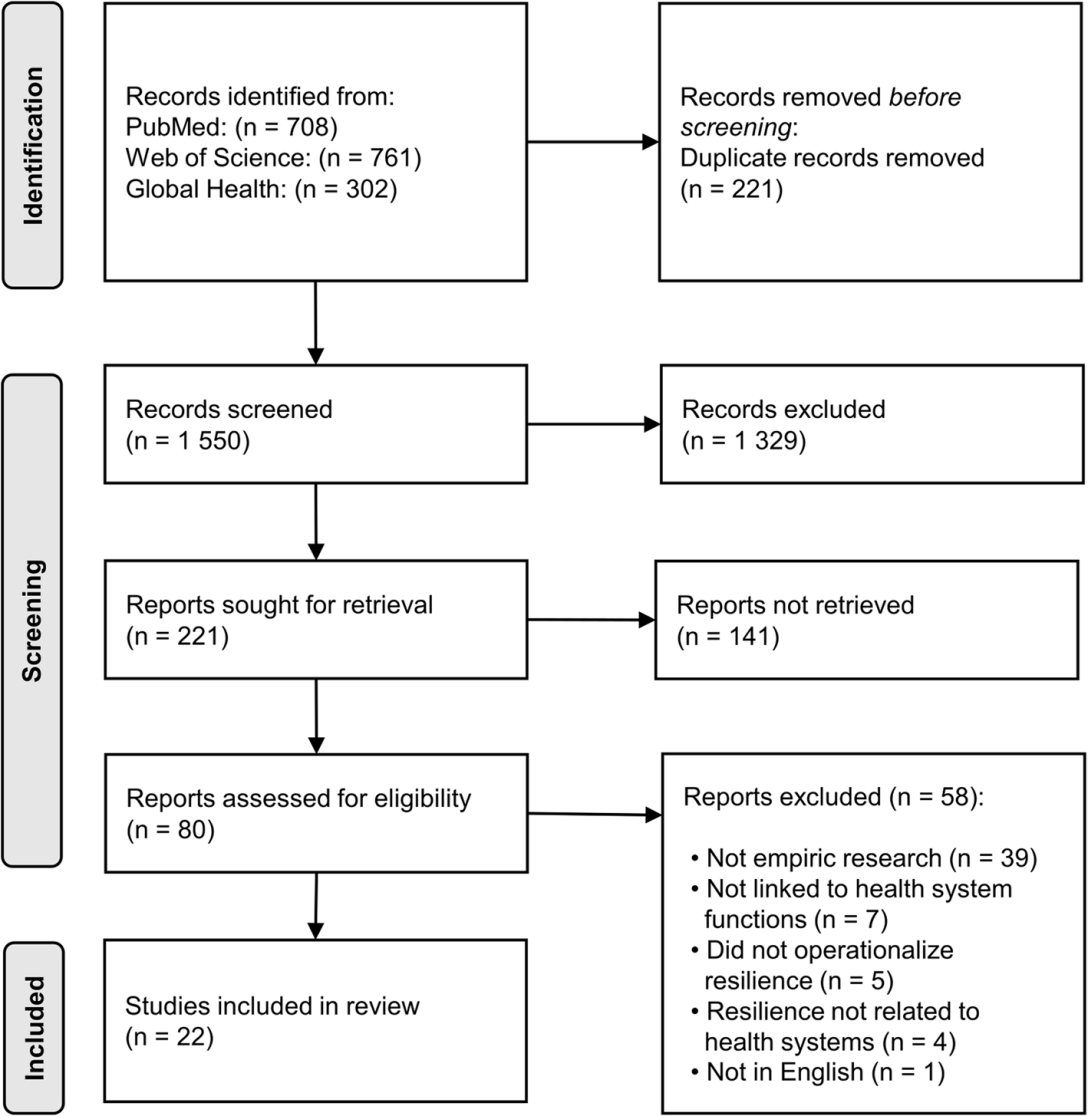
PRISMA Diagram showing the articles excluded during each step of the screening process

Variations on the search terms health systems OR healthcare system AND resilience OR resilient, and health systems strengthening AND resilience OR resilient were used to capture the most relevant studies (Additional file 1). The following electronic databases were searched in January 2021: PubMed, Web of Science, and the Global Health database. After removing duplicates, 1 150 articles were identified (Fig. 1). Articles were screened by title, abstract and full text by the first author, who consulted with the last author if the eligibility of a study was unclear. A total of 22 studies were included in the analysis.

After screening, the articles were reviewed to identify and extract detailed information on the strategies for resilience. Strategies were defined as actions or plans that the authors reported were supportive to resilience or were implemented to support one or more author-defined resilience capacities [4]. Data was also extracted on study design, methods, geographic area, country context, health system components under study, type of shock or crisis, and any resilience framework(s) used in the study design. Findings from the studies on resilience capacity, maintaining core functions, the system’s reorganization, or lessons learned during the crisis were also extracted. No new data were generated or analysed in support of this research.

The data were analyzed by narrative summary. The resilience strategies and the findings were compared to each other by identifying common ideas and identifying key words in the text. Strategies were then grouped by similarity into thematic areas that arose from the process of identifying common thematic areas. Next, the thematic areas were refined by comparing strategies between thematic areas to confirm the grouping, and then by rereading and drafting a definition for each thematic area that encompassed all strategies within the theme.

## Results

Twenty-two studies were included in the final analysis (Additional file 2: Table 2). Four papers were evidence synthesis papers; nine of the empirical papers were also included in the synthesis papers. Fifteen studies were conducted in low- or middle-income countries. Eighteen studied shocks, the sudden events that begin outside the health system and impact the resources available to the system and the demand on the system. Three articles studied stresses, which are the internal challenges that the health system regularly experiences. One article studied both. The shocks included extreme weather events, floods, refugee and migration crises, the 2014–2016 West Africa Ebola outbreak and other infectious disease outbreaks, financial crisis, and conflict. All three stress studies looked at decentralization processes in the health system. Fifteen papers concentrated on the health system at national level while six papers looked at sub-regional or district levels.

Nine main thematic areas were identified in the resilience strategies that were used (Table 2). Governance and financing, Leadership, Surveillance, and Human Resources were the thematic areas most frequently referred to as important for sustaining and building resilient health systems. A list of all resilience strategies that were used or recommended in the studies is given in Additional file 3: Table 4.

**Table 2:** Description of the main thematic areas, including the number of articles that were identified per area

Main thematic areas identified	Description of thematic area
Use of community resources (*n* = 4)	Use of community-based healthcare delivery (community health care workers) or voluntarily engagement and mobilizing of community members
Improved governance and financing (*n* = 7)	Governance and financing includes strategies on budgeting, reforms, policy making, decision-making process, national and international funding and financial accountability and transparency
Empowered middle-level leadership (*n* = 6)	Leadership refers to senior and middle managers in the health sector including roles, responsibilities, management style, values, trust, and ability to create reflective spaces
Improved surveillance systems (*n* = 6)	Surveillance includes health information systems, messaging and collaborating with other systems and the public to track necessary information
Strengthened human resources management and capacity (*n* = 5)	The health workforce including number of staff, task-shifting, flexible working modes, motivation, training including on-the-job training and involvement and participation of staff in processes and decision-making
Improved communication and collaboration (*n* = 4)	Communication includes internal and external communication and collaboration with partners in the health system and with other sectors, including private–public partnerships
Improved preparedness (*n* = 3)	Preparedness for crises include planning, response plans, checklists, protocols, and disaster risk analysis and management
Strengthened organizational capacity and learning (*n* = 4)	Organizational capacity and learning include the ability of the health system and actors to organize, function and learn within a certain context and with the resources available
Re-emphasized health systems strengthening (*n* = 4)	Health system strengthening include universal health coverage and core capacities of health security and International Health Regulations

### Use of community resources

Plans and actions to engage community members and involve and use community health workers [6–9] were described as ways to support the functioning of the health system during crises and improve the community’s trust in the system for future shocks. Community capacity strategies were primarily highlighted in crises such as infectious diseases and natural hazards in low- and middle-income countries (LMICs).

A scoping review of extreme weather events in Asia–Pacific concluded that utilizing communities as a resource was an asset to the health workforce. For example, community health workers were an additional resource that could be mobilized to support vaccination campaigns, conduct surveillance activities, and deliver basic first-aid kits [7]. Two articles on the Ebola outbreak in West Africa showed the communities’ important role in the response: investments in community-based health care contributed to lifesaving treatments through the delivery of core services for child pneumonia and diarrhoea during the Ebola crises, when facility-based health services were impacted by crisis; and that forming community-based surveillance teams led to improved communication with and increased trust in health authorities. Linking community services to first-level health facilities resulted in more effective risk communication and actions to reduce risks [6, 9]. Saulnier et al. report that in terms of pregnancy and childbirth care during floods in Cambodia, greater involvement in decision-making at local level could have facilitated community members’ own power to advocate for their preferred source of care during floods [8]. Further involvement in decision-making for care might improve resilience by improving the community’s capacity to adapt and transform when experiencing a crisis.

### Improved governance and financing

The capacity of national governments to develop and manage policies and partnerships for health systems, to have financial accountability mechanisms in place, and to foster political commitment to health were stressed as important for building resilient health system [7]. The whole of government—together with the health system—need to prepare, be aware, be flexible and be able to adapt in crises. In contexts with high levels of stress or fragile and conflict-affected settings, strong local governance was a more salient factor for the continuity of health services because of its distance from national-level challenges and closeness to local populations; this included access to health resources and humanitarian funding [10]. In both LMIC and high-income country (HIC) settings and in situations of conflict and chronic stress, health system ‘software’, such as motivation and management capability, and a strong emphasis on learning and recovery were seen as important for building and nurturing health systems resilience [11].

In the article on extreme weather events in Asia–Pacific, inadequate funding, a lack of budgeting for healthcare-related activities during disasters and challenges in navigating bureaucratic processes for additional funding were seen as weakening resilient health systems [7]. Lessons learned from organizational change and everyday health system resilience in Cape Town, South Africa supported micro-practice governance as a practice to respond to stress in the health system and to nurture organizational resilience capacities [12]. Odhiambo et al. used three health systems resilience outcome definitions (maintaining function, improving function, and achieving health system targets) and concludes that the best definition of resilience in a conflict setting is the definition on improving function [10]. Realistic and clear health systems targets were considered important for measuring resilience progress as well, as when mobilizing resources such as humanitarian funds to the health system.

A generic checklist to improve health systems resilience was developed for infectious disease outbreaks and natural hazards while studying experiences from Bangladesh. Even so, it concludes that the checklist cannot be maintained without also addressing structural, economic, and political barriers, such as social determinants of health, which is a task for the government to address [13]. A public health framework for promoting resilience based on experiences from Canada stressed the importance to enhance a culture of preparedness including ethics and values [14]. At the same time a scoping review on what makes health systems resilient against infectious disease outbreaks and natural hazards reports on the importance of what happens after a crisis in terms of recovery and lessons learned. More action-oriented steps, for example towards recovery and learning at the national level, is important for health actors including governments [11]. Experiences from a systematic review on resilient health systems in Sub-Saharan Africa showed the importance of governance capacity for health system strengthening in general, including financial accountability [15].

### Empowered middle-level leadership

Leadership refers to operational or administrative management. Decentralising decision-making, participation, and accountabilities to lower levels were described as important in terms of adjusting and adapt in a crisis [14]. Systems showed greater flexibility when decisions were made closer to operational levels of the health system. Clearly articulated management roles with mechanisms to hold them accountable and strong communication were also stressed as crucial when the leadership needed to be aware of chronic stress and create for example spaces of reflection for staff [16]. In particular, decentralization of operational decision-making and the role of middle managers were highlighted in systems experiencing chronic stress due to decentralization reforms [17, 18].

The decentralization of decision-making to lower system levels was an important organizational capacity for resilience. For example, delegating decisions from central bodies at the United Nations Relief and Works Agency for Palestine Refugees in the Near East (UNWRA) to field offices was reported as particularly useful for maintaining continuity of services during an influx of Palestinian refugees from Syria in Lebanon and Jordan [17]. A study of a district health system exposed to chronic stress in Kenya reported that managers that managed to demonstrate good values and communication helped staff become less resistant to implementing response to different stresses. The authors describe this as an important example of how organizational capacities for everyday resilience might be strengthened [16]. An additional two studies exploring chronic stress in Kenya and South Africa at the district level also describe the importance of middle management for everyday resilience and supporting reflection and learning [12, 18].

### Improved surveillance systems

Improving surveillance systems was highlighted as a key strategy for creating resilience for infectious disease crises, such as Ebola in the Southern Africa region and in West Africa, and as a major reason for sustaining resilience during late stages of crisis response [15, 19, 20].

During the 2014–2016 West Africa Ebola outbreak, new surveillance system structures in Liberia were introduced by international partners but were operated by the Ministry of Health. The authors suggest that the better integration observed during the later stages of the response were linked to the new surveillance structures [20]. Improved health information systems and data collection were highlighted as a main resilience strategy when looking at experiences from different shocks in Europe (financial crisis and migration crisis), West Africa (the 2014–2016 West Africa Ebola outbreak) and climate-related crises in the Philippines and Haiti [19]. Ayanore et al. suggest that investments in new and improved surveillance systems and surveillance capacity are crucial for the Southern Africa region to improve health system preparedness for health threats [15].

### Strengthened human resources management and capacity

Across low-, middle-, and high-income contexts and multiple types of shocks, a committed, adequately sized, flexible, and trained workforce were seen as crucial for creating and sustain resilience [13, 15, 16, 21]. The resilience of UNRWA health system during the Syria crisis suggests that staff commitment was important in maintaining service continuity and quality to Palestinian refugees [21]. The article on chronic stress to the health system at sub-national level in Kenya due to rapid devolution concludes that a new human resource advisory committee facilitated stakeholders to meet and advise on human resource issues, such as promotion and in-service training [16]. Lessons learned for the US in their domestic Ebola response showed that confidence was built and stress alleviated when staff were involved in developing infection prevention protocols [22]. Based on the findings of their studies, other authors suggested that appropriate training needs to include longer-term planning for what happens after the emergency stage is over and that continual on-the-job training for health professionals is a preferred option compared to long term offsite training of the health workforce, especially during emergencies and in the LMIC context [15].

### Improved communication and collaboration

In addition to engaging communities as a resource, transparent communication and good collaboration within the health system and with other groups and actors outside the health system were given as resilience strategies. This included strategies such as establishment of coordination fora and using a wide array of service providers to enhance public trust in the health system and in public health matters. The UNRWA in Syria managed to collaborate with government, local and international partners (for example on vaccines and releasing medicine stock from the port) to maintain service provision for Palestinian refugees [17]. Furthermore, the influx of Syrian refugees in Lebanon due to the Syrian conflict led to an integrated response plan with many stakeholders which included a wide array of service providers from private, public sectors and NGOs and multiple sources of funding [23]. Ling et al. report that in the late Ebola response in Liberia the approach became more integrated and in order to facilitate coordination with health and non-health actors a central level coordination forum was established [20]. Wang et al. concluded that there is a need to strengthen communication with the public, enhance public trust in the health system, and better engage with societies in the countries to tackle public health emergencies [24].

### Improved preparedness

Preparedness by health actors was seen as key for resilience, to be aware and to prepare for crises. Integrated response plans with many stakeholders, emergency plans, checklists, protocols, and the importance to relate to and liaise with the political system were highlighted in for example the refugee crises due to the Syrian conflict [17, 23] but also in a LIC such as Bangladesh which is often struck by infectious diseases and natural hazards [13]. Meyer et al. developed a resilience checklist for infectious disease outbreaks and natural hazards based on experiences from Bangladesh’s health system, and conclude that such checklists are an important tool for preparedness since they can facilitate what to do if such a crisis situation occurs [13]. Dealing with refugees coming from Syria in Lebanon and Jordan, measures to make sure to integrate refugees into the national health system and make sure they were acknowledged by the host country government, were seen as ways to prepare for service delivery to refugees [17, 23].

### Strengthened organizational capacity and learning

Organizational functioning and the ability of health actors to learn and adapt within the national context and with existing resources are particularly important for building resilient health systems in situations with chronic stress and in contexts where resources are scarce [1, 16, 18, 25]. The examples from Kenya and South Africa on stress and everyday resilience concludes that it is most important to look at capacities that underpin strategies since they are the capacities that can strengthen health system responses to everyday challenges [16, 18]. Enhancing the strategic, continuous learning for Ministries of Health is given as a useful strategy for building resilience in LMICs [13]. Barasa et al. also suggests that it is important to nurture health systems resilience by looking at everyday challenges in health systems and invest in processes that promote the adaptive capacity of health systems [1]. However, these strategies described were only studied in relation to situations of chronic stress rather than shocks.

### Re-emphasized health systems strengthening

Universal health coverage was seen as an important resilient measure for protecting the most vulnerable in crises in terms of service delivery [26]. Furthermore, health actors’ knowledge and capacity around health security and International Health Regulations (IHR) were recommended to be part of national health system strengthening efforts including training of staff [24]. In Ireland the economic crisis in 2007 affected the health system, and it was concluded that the most positive step for building a resilient health system in future was the country’s first step to outline universal health coverage through a universal health insurance system [26]. The article on lessons from the domestic Ebola response in the US conclude that every facility should at minimum be able to identify, isolate and stabilize until referred to specialized treatment hospitals [22]. Wang et al. emphasize health system strengthening in general and specifically the integration between health system strengthening and health security when working towards resilient health systems, based on experiences so far from the COVID-19 crisis in a few countries [24].

## Discussion

The results from this review identifies examples of strategies and ideas used or recommended within the thematic areas. The findings emphasize the importance of improved governance and financing, empowered middle-level leadership, improved surveillance systems and strengthened capacity and management of human resources. These thematic areas are closely linked to broad health system functions.

Strategies to empower leadership, in particular middle-managers and in combination with good management skills, may be useful for building resilience. Middle managers are seen as key for nurturing resilience since they are close to the frontline workers and could facilitate support, reflection, and learning [18]. These findings were primarily observed in studies on health systems in situations of chronic stress rather than shocks, which focused more on maintaining management systems for human resources and surveillance. Tied to the importance of middle managers was the emphasis on health systems as learning organization to be resilient, an area of growing importance for resilience research [25]. Adaptive resilience and the ability to adjust and be flexible may be more important than planned resilience (preparedness) when the crisis comes, especially in LICs where resources are scarce and it is therefore difficult to prepare for everything [1]. The literature on health systems resilience also suggests that more thinking needs to go into the adaptive capacity [27]. Lessons learnt exercises are important for the health system to adjust and prepare itself before the next crisis. Strategies to learn from previous experience that address the different functions, areas, and levels of the health system may be one way to strengthen health system resilience during recovery from a shock.

Similar thematic areas which were identified in this study were furthermore suggested in a policy and planning meeting in 2020 for the WHO’s Africa region focusing on building health system resilience [28]. Collaboration between sectors, preparedness using an integrated approach (for example integrated response plans) and organizational learning are the thematic areas very close to the conclusions made in this WHO Africa policy meeting. Since Africa has experienced many crises including the Ebola virus, the conclusions made and strategies used in many of the African countries are potentially a valuable addition to global discussions on resilience strategies, for example the emphasis that were put on collaboration. The focus on universal health coverage and health security are emphasized by Tumusiime et al. as agendas needed to be brought forward in the situation today with the COVID-19 pandemic [28]. The largest challenge in the Africa region was seen as the limited adaption from past experiences as some systems are facing the same problems repeatedly. In light of this, if there will be an increased focus on learning from crises this will most likely result in a better understanding about resilience strategies and what works.

The strategies in this review are similar to strategies that were identified in Thomas et al*.*’s report on health system resilience concepts and strategies [4]. Areas such as competent and responsive leadership, adequate surveillance, and effective collaboration and coordination were identified as potentially useful strategies in both sets of findings. Some areas like organisational learning and health system strengthening were emphasized more in this study, likely due to the results of this study being presented as thematic areas rather than health system functions. The alignment between results is not surprising, as the report also reviewed some of the same literature on health system resilience. However, the alignment does suggest that the strategies in the review are likely to be useful and of interest for further exploration and implementation, since the report also included new evidence on strategies from the Covid-19 pandemic and from additional case studies.

The strategies and ideas are in principle relevant for all country contexts. But what a health system should emphasize, in what order, and how a health system should design its own detailed strategies depend to a large extent on the country’s context. This includes the governance context, the available resources for health, the strengthening of the health system prior to a crisis, and the type of crisis that the system is exposed to [13, 16]. However, two distinct contextual areas emerged from the review. First is the importance of good governance and accessible health resources in building resilience in fragile and conflict-affected contexts. It may be that developing strategies targeted to governance and resource availability would be most beneficial in these settings, an idea that has been recognized in earlier resilience frameworks [11]. Still, weak governance and limited resources are common problems in fragile and conflict-affected states regardless of their exposure to shocks [29–31]. This is related to the second contextual area identified in the review: the difference in strategies between stresses and shocks. Strategies to strengthen organizational capacity, learning and nurturing resilience were mainly identified in the context of stress, along with strong management ability to support staff under pressure and enhance flexibility. Strategies identified mainly in shocks, where events need to be managed quickly, tended to focus on promoting having strong system functions in place, such as surveillance systems and human resource support, to manage such crises and support resilience.

The results of the study should be interpreted with several limitations in mind. The analysis presents a descriptive summary of resilience strategies. It is not possible to interpret the impact of the strategies from the descriptive analysis, and the findings of this paper rely on the articles’ conclusions that the strategies supported resilience. A further causal analysis of the strategies would be a useful addition to knowledge on strategies for resilience. The health system context, the governance context, the study objective, the methodology, and resilience frameworks used differ as well as crisis studied and whether it is a LMIC, HIC or global focus. For instance, all studies on community engagement and involvement were set in LMICs and strategies could differ in HICs. Therefore, it is difficult to make some certain general conclusions. Other limitations include a lack of sources from more databases, inclusion of grey literature and information from websites of international organizations, since important research could have been missed that would be valuable to the research question of this study. Such sources could also have provided a better understanding of practitioners working in this field, for example from evaluations. The articles highlighted several thematic areas including strategies in their research results. Some of the strategies could also belong to several thematic areas. However, in presenting the results from this study a strategy is only presented once, within the thematic area that was considered most appropriate. A second reviewer would have contributed to a more thorough research process in the screening and study selection process and reduce the risk of paper selection bias which could impact study findings. Finally, the search strategy was developed without the support of a librarian. It is possible that additional articles would have been identified if the search strategy had been validated by a librarian.

## Conclusion

The strategies for building health systems resilience that were emphasized were those that improved governance and financing, empowered middle-level leadership, improved surveillance systems, and strengthened human resources. We observed a difference in strategies for stressed health systems—strategies related to organizational capacity, learning and management ability—and shocks, where strategies focused primarily on strengthening system functions. While this study elaborates on what strategies have been used or are recommended as important in building resilience, it has not been possible to analyse in depth why and how they build resilience. The mechanisms that build health system resilience still need to be better understood, so that appropriate strategies can be taken up and implemented by practitioners in health systems in varied contexts. Doing so may build resilient health systems that have the capacity to adapt and to learn from both stresses and shocks.

## Supplementary Information

**Additional file 1.** Database search strategies. Detailed search strategies used in the databases.

**Additional file 2: Table 2.** Summary of included studies. Overview table of all papers included in the study, with author name, year, objective, design and method, context, and summary of main findings.

**Additional file 3: Table 4.** All resilience strategies that were reported, within the identified thematic areas. List of all strategies identified in the paper, with thematic area, strategy, geographic area, scope of health system, and type of crisis. 

## Data Availability

Not applicable.
