# Supplementary material for: Health systems resilience in practice: a scoping review to identify strategies for building resilience
Source: BMC Health Serv Res. 2022 Sep 19;22:1173. doi: 10.1186/s12913-022-08544-8 (PMC9483892; doi:10.1186/s12913-022-08544-8)
Supplement: Supplementary file 1 — Additional file 1. Database search strategies. Detailed search strategies used in the databases. [file 12913_2022_8544_MOESM1_ESM.docx]

**Appendix 1:** Database search strategies

**PubMed**

”health system” OR ”healthcare system” OR “health systems” OR “health care system” OR “health care systems”

Resilience OR resilient OR resiliency

(”health system” OR ”healthcare system” OR “health systems” OR “health care system” OR “health care systems”) AND strength*

1 AND 2

2 AND 3

”mental health” OR psychology OR psychologic OR psychological

4 OR 5 NOT 6

4 OR 5 NOT 6 Timespan 2013-2021

**Web of Science**

1. TS=(health system” OR “health systems” OR “healthcare system” OR “health care systems” OR “health care system” OR “health care systems)

2. TS= (resilience OR resilient OR resilie*) OR AK=(resilience OR resilient OR resilie*) OR KP= (resilience OR resilient OR resilie*)

3. TS= (( “health system” OR “health systems” OR ”healthcare system” OR “healthcare systems” OR health care system“ OR ”health care systems”) AND strength*)

4. #1 AND #2

5. #2 AND #3

6. TS=(“mental health” OR psychology OR psychiatry)

7. (#4 OR #5) NOT #6

8. #7 timespan 2013-2021

**Global Health Database** (Ovid)

(“health system” or “health systems” or “healthcare system” or “healthcare systems” or “health care system” or “health care systems”)

(resilience or resilient or resilie*)

((“health system” or “health systems” or “health care system” or “health care systems” or “healthcare system” or “healthcare systems”) and strength*)

1 and 2

2 and 3

(“mental health” or psychology or psychiatry)

(4 or 5) not 6

limit 7 to (English language and yr=”2013-2021”)
