# Supplementary material for: Health systems resilience in practice: a scoping review to identify strategies for building resilience
Source: BMC Health Serv Res. 2022 Sep 19;22:1173. doi: 10.1186/s12913-022-08544-8 (PMC9483892; doi:10.1186/s12913-022-08544-8)
Supplement: Supplementary file 2 — Additional file 2: Table 2. Summary of included studies. Overview table of all papers included in the study, with author name, year, objective, design and method, context, and summary of main findings. [file 12913_2022_8544_MOESM2_ESM.docx]

**Table 2.** Summary of included studies

| **First author, year** | **Study objective** | **Study design and method** | **Context** | **Summary of main findings** |
| --- | --- | --- | --- | --- |
| **Ramesh-shanker**  **2021** | To map and describe how existing literature has operationalized national health resilience to extreme weather events (EWE) in Asia-Pacific | Scoping review of peer-reviewed and grey literature to identify assets and/or gaps to resilience | EWEs in Asia-Pacific, focusing on Philippines, India, and Thailand | Sustainable health system financing an asset for resilience to EWEs, the 'what and how' for health system financing should be a forefront concern for policy practitioners. Leveraging communities as a resource for supporting service delivery, community education for self-management, and volunteer mobilization can promote resilience. |
| **Alameddine**  **2019** | To study the ability of the UNRWA to deliver health services to Palestinian refugees in Syria during the ongoing crises | Qualitative case study during 2017 using 35 semi-structured interviews and group model building | Palestinian refugees in Syria; system-level challenges and resilience capabilities | Strategies for staff commitment, organizational flexibility, decentralised operational decision-making and internal and external collaboration and communication can help maintain continuity of services. |
| **Ling**  **2017** | To review the evolution of Liberia's health system response to the 2014-15 Ebola epidemic from the perspective of global, national, and local actors involved | Qualitative study using 108 semi-structured interviews, in-depth interviews, and focus group discussions between July and September 2015 | Seven counties in Liberia during the 2014-2016 Ebola-crisis | Positive changes and progress were related to system-level strategies in surveillance, communication and coordination and collaboration, and were prioritised at global and national level efforts. |
| **Kagwanja**  **2020** | To examine the challenges experienced by the health system at sub-national level in Kenya using an ‘everyday resilience’ lens, with a view to add to the understanding of health system resilience | Participatory approaches, in-depth interviews, informal observations, informal interviews, and reflective practices in an embedded learning site between September 2017 and November 2018 | Devolution from a centralized national health system to a decentralized governance system in Kilifi district, Kenya | Organizational capacities for everyday resilience that counter challenges include governance arrangements that empower actors to make transformative actions that enhances contextual capacity; networks between health managers and local politicians; spaces and opportunities for reflective exercises to nurture cognitive, contextual and behavioural capacities; and managers demonstrating good values and good communication. |
| **Thomas**  **2013** | To develop a framework for assessing the resilience of health systems in terms of how they adjusted to economic crisis | Mixed methods study semi-structured interviews with system stakeholders and secondary health information data between 2008 and 2012 to understand the depth of recession and dimensions of response | 2008 financial crisis in Ireland | The system displayed financial resilience by protecting access to services and some entitlements. The system adapted by reducing wages and fees and implementing moratorium on recruitment, with limited impact on services, and transformed by prioritizing universal health coverage through a plan for a universal health insurance system. |
| **Meyer**  **2018** | To identify and integrate lessons from Ebola in West Africa into an actionable checklist that can improve health sector resilience | Two focus group discussions and 73 interviews with individuals involved in the domestic US Ebola virus disease response between 2016-2017 | The domestic response to the Ebola virus disease outbreak in Atlanta, Dallas, New York and Omaha | Healthcare facilities needed to adapt to fulfil their responsibility for the response. Facilities needed to develop the capacity to identify, isolate and stabilise cases, improve laboratory capacity, develop health information campaigns and public outreach to help with fear and stigmatizing, and further engage health staff in protocol development and community outreach. |
| **Gilson**  **2020** | To examine how health managers and staff in one local health system in Cape Town responded to centrally imposed processes of organisational change and primary health care service improvement | Multiple types of observations, interviews and secondary data analysis to track implementation and system experience between 2017-2018 to explore local health system change over time | The political decentralization process in Cape Town, South Africa | Strengthening micro-governance processes and interventions and the leadership practices that underpin everyday decision-making can support resilience, particularly among the critical group of mid-level managers. |
| **Odhiambo**  **2019** | To examine three current health system resilience definitions using real time data from South Sudan to develop a data driven understanding of resilience | Assessing the relationship between a stress index derived from health system data and a resilience index from maternal, newborn, and child health coverage indicators from household surveys | South Sudan, maternal and child health in South Sudan following two years of protracted conflict. | Resilience can be measured as the ability to improve functioning during protracted periods of stress, in conjunction with improved local governance, access to health resources, and humanitarian aid. Maintaining functioning may be a useful measure for resilience in health systems facing acute stress. |
| **Meyer**  **2020** | To develop a checklist to be adapted and used in a broad set of countries as a component of ongoing processes to ensure that health actors, institutions and populations can respond effectively to infectious disease outbreaks and natural hazards while also maintaining core healthcare services | Development of a checklist from a scoping review of peer-reviewed and grey literature, comparison with the WHO Joint External Evaluation tools, and key informant interviews of stakeholders | Infectious disease outbreaks and natural hazards, from experiences in Bangladesh | Areas identified as important for resilient health systems included infrastructure, financing, reducing barriers to care, leadership, communication and collaboration, surge capacity, risk communication, the health workforce, and infection control. Improving these areas requires strong processes, mechanisms, and policies from health system actors. |
| **Khan**  **2018** | To describe the essential elements of a resilient public health system and how the elements interact as a complex adaptive system | Qualitative study of six focus groups across Canada using a Structured Interview Matrix (SIM) | Public health emergency preparedness in urban, urban-rural, and rural areas of Canada | The framework identified 11 essential elements that interact within complex adaptive systems to create resilience, including clear leadership; integration of public health with non-health sectors; planning using a dynamic collaborative process that includes the community; timely information for awareness and action; clear, consistent messaging across networks and to the public; evaluation and learning; and strong core principles to guide policy and practice. |
| **Ammar**  **2016** | To assess the resilience of the Lebanese health system in the face of an acute and severe crisis and in the context of political instability | A case study using a literature review of resilience and analysis of secondary health system data to document the impact of refugee crisis and the health system response in Lebanon | Syrian refugee influx in Lebanon between 2011 and 2013 | The Lebanese health system sustained its performance during the crisis, including retaining governance and financing, and maintaining service delivery for both refugees and Lebanese citizens. Performance was supported by networking with multiple partners in the health sector, mobilizing support from regional and global partners, adequate infrastructure, sufficient supply of human resources, and integration of refugee health care within the national health system. |
| **Siekmans**  **2017** | To examine the value of a community-based health system in ensuring continued treatment of child illness during the outbreak and the role that community health workers (CHWs) had in Ebola prevention activities | Mixed methods using child health data from CHW and health facility records, a survey of 60 CHWs, and 16 focus group discussions with CHWs, health facility staff, and project staff between 2014-2015 | The 2014-2016 Ebola virus disease outbreak in rural areas of Liberia | Community Health Workers were a trusted source of advice and in Ebola prevention education. Investments in community-based health service delivery contributed to continued access to life saving treatment for child pneumonia and diarrhoea during the Ebola outbreak, making communities more resilient when facility-based health services were impacted by the crisis. |
| **Wang**  **2020** | To examine the health systems resilience of selected countries and analyse their strategies and countermeasures in response to Covid-19 pandemic | A cross-sectional study using secondary data on COVID-19 deaths, interventions and strategies, and International Health Regulation (IHR) and global health security index scores | The first 6 months of the COVID-19 pandemic in Japan, Iran, South Korea, UK and USA | Health system strengthening and health security efforts should be integrated and pursued in tandem, as mutually reinforcing approaches to developing resilience. Communication with the public, public trust in the health system, and engagement with society to tackle public health emergencies should be strengthened. |
| **Barker**  **2020** | To adapt and apply existing theoretical community engagement frameworks to health system resilience | Qualitative study using 92 in-depth interviews and 16 focus group discussions with health system stakeholders | The 2014-2016 Ebola virus disease outbreak in Liberia | Community engagement was a crucial contributing factor in addressing the Ebola epidemic in Liberia by increasing trust in the health system and improving communication. Communities should be continually engages as partners rather than beneficiaries. |
| **Gilson**  **2017** | To explore the need for and nature of everyday resilience within health systems, considering the routine challenges they face and the strategies employed to address them | Embedded learning site using document reviews, 29 in-depth interviews, group discussions, observations, and reflective practice between 2017-2018 | Decentralization in three districts in Kenya and South Africa | Everyday resilience requires empowering leadership beyond stable governance, including the important role of middle managers and their managerial strategies and organisational capacities. Closer attention should be paid to software of health systems, governance, and leadership. |
| **Alameddine**  **2019** | To advance the understanding of resilience of health systems by interrogating the appropriateness of a capacity-oriented resilience framing via a case study of UNRWA | A qualitative research design with 62 key informant interviews with UNRWA health system professionals in Lebanon and Jordan between 2016-2017 | Palestine refugees from Syria in Lebanon and Jordan, and the UNRWA health system | The UNWRA system was able to absorb, adapt, and transform by broadening collaborations, reconfiguring staff roles, and revising the service packages that were provided. |
| **Saulnier**  **2020** | To understand how community management of pregnancy and childbirth care during floods is contributing to the system's capacity to absorb, adapt or transform as viewed through a framework on health systems resilience | A qualitative study with 8 focus group discussions and 17 semi-structured interviews with community members in 2018 | Pregnancy and childbirth care during floods in Cambodia | There was limited involvement of the community in decision-making for care, a lack of community ownership in the health system and a power imbalance between the community and the health system. Further involving the community in decision-making for care could have helped to improve the health system's resilience by creating room for the community to adapt and transform when experiencing floods. |
| **Hanefeld**  **2018** | To advance the discussion on how health systems can respond to shocks and therefore, be more resilient, by looking at four recent shocks | Case studies for four shocks and contexts, using existing literature | The 2008 financial crisis and the 2015 refugee crisis in Europe; climate change in low- and middle-income countries; the 2014-2016 Ebola virus disease outbreak in West Africa | Five critical dimensions supported resilience in the case studies: the system's ability to gather information and make decisions with it; investing and mobilizing resources to fund a response; the workforce to plan and implement a response; the governance of the system as a whole; and the values that shape the response at the system, individual, and community levels. |
| **Nuzzo**  **2019** | To identify recurring themes and capacities needed for health system resilience and any existing implementation frameworks that highlight these capacities | Scoping review of literature on health security, health system strengthening, and quality importance | Global, in relation to outbreaks and natural hazards | The themes included developing policies for determining what level of care will be delivered when the level of demand exceeds existing resources, planning for post-event recovery, and a commitment to quality improvement that ensures integration of lessons learned. |
| **Naimoli**  **2018** | To explore the potential of health ministries to become learning organisations to help foster resilience | Multi-stage, iterative literature searches to identify resilience and learning organization literature and learning organization conceptual framework, complemented by expert opinion | Health ministries in low- and middle-income countries! | Health ministries face substantial challenges in trying to realise their learning potential, including a lack of models on sustained learning in health sectors in LMIC. Management and measurement skills for learning can be enhanced by technical assistance, incorporating a learning plan into national health strategies, embedding learning into leadership, and creating a supportive learning environment. |
| **Ayanore**  **2019** | To examine the literature on health workforce, surveillance, and health governance issues for health systems strengthening | Systematic review of publications on health workforce, health information systems, and leadership and governance | Health systems in Sub Saharan Africa | Having an adequate health workforce, health surveillance, and health leadership and governance are identified as major factors in strengthening health systems in Sub-Saharan Africa. Important strategies for resilience include task shifting, performance-based payments, and results-based financing, data quality improvement techniques, policy shifts that prioritise training health care workers, investment in robust and cost-effective surveillance capacity, and creating financial accountability in health financing and governance. |
| **Barasa**  **2018** | To review the empirical literature from both the health sector and other sectors to synthesize evidence on organizational literature | Systematic review of organizational resilience literature | Global | Important to recognize health system resilience as a function of planning and preparing for shocks and adapting in response. Factors that influence the resilience of an organisation are material resources including finances, adequate preparedness and planning, strong information management, redundancy, good governance process and leadership practices, the organisational culture, human capital, and social networks and collaboration |
