# Supplementary material for: Health systems resilience in practice: a scoping review to identify strategies for building resilience
Source: BMC Health Serv Res. 2022 Sep 19;22:1173. doi: 10.1186/s12913-022-08544-8 (PMC9483892; doi:10.1186/s12913-022-08544-8)
Supplement: Supplementary file 3 — Additional file 3: Table 4. All resilience strategies that were reported, within the identified thematic areas. List of all strategies identified in the paper, with thematic area, strategy, geographic area, scope of health system, and type of crisis. [file 12913_2022_8544_MOESM3_ESM.docx]

**Table 4.** All resilience strategies that were reported, within the identified thematic areas

| **Thematic area identified** | **Resilience strategies used or recommended within the thematic area** | **Geographic area** | **Scope of health system** | **Type of crisis** |
| --- | --- | --- | --- | --- |
| *Use of community resources* | - Using community health workers (CHW) to support service delivery  - Community education for self-management of health needs  Mobilizing volunteers | Asia Pacific | National health systems | Extreme weather  events |
|  | - Linking community services to first level facilities to maintain continuum of care for child health  - Mitigating negative impacts of crises by recruiting CHWs as trusted sources of advice and providers of prevention education | Liberia | National health system, rural areas | Ebola outbreak |
|  | - Engaging with communities: treating communities as partners not beneficiaries  - Formalize partnerships for community-based surveillance before a crisis  - Creating community engagement platforms and public trust ahead of crisis | Liberia | National health system | Ebola outbreak |
|  | - Greater involvement of decision-making at local levels by communities  - Improving two-way flow of information between the community and health services | Cambodía | Health system at district level | Floods |
| *Governance and financing* | - Available and accessible budget for crises  - Deciding on how and for what the health system should be financed | Asia-Pacific | National health system | Extreme weather  events |
|  | - Using governance principles of sharing, listening, thinking, and reflecting together to develop innovations in micro-practice of governance  - Giving equal attention to top-down and bottom-up approaches to governance | South Africa | Health system in Cape Town | Chronic stress due  to decentralization process |
|  | - Improving local governance and access to health resources  - Creating realistic, clear, and transparent health targets and indicators to measure achievement of health system resilience | South Sudan | National health system | Conflict |
|  | - Addressing social determinants of health | Bangladesh | National health system | Infectious disease outbreaks; natural hazards |
|  | - Establishing a culture of preparedness knowledge and action, including risk analysis, surveillance and monitoring, resources, workforce capacity, and networking | Canada | Local and regional public health facilities | Emergencies and  disasters |
|  | - Developing policies for determining the level of care to be delivered when the level of demand exceeds existing resources  - Planning for post-event recovery  - Committing to quality improvement that ensures integration of lessons learned  - Maintaining critical infrastructure and transportation  - Ensuring timely and flexible access to emergency and crisis funding  - Establishing a clear and flexible command structure prior to an event | Global | National health systems | Infectious disease outbreaks and natural hazards |
|  | - Creating financial and administrative accountability in health financing and governance  - Establishing public-private partnerships  - Developing polices that address broader determinants of health  - Continued political commitment  - Using results-based financing | Sub-Saharan Africa | National health systems | Primarily infectious diseases |
| *Leadership* | - Clearly designating roles and responsibilities  - Clarifying existing leadership and governance infrastructure | Asia-Pacific | National health systems | Extreme weather  events |
|  | - Decentralising operational decision-making to operation managers in the respective area, clinic, or field office | Palestinian refugees in Syria | UNRWA health system for Palestinian refugees | Syria conflict |
|  | - Managers demonstrating good values and good communication to reduce resistance of staff to implement a response to stressors  - Creating opportunities and spaces for reflection | Kenya | Subnational health system | Stress due devolution process |
|  | - Importance of mid-level health managers, positioned between centre and the frontline, in supporting reflection and learning | South Africa | Health system in Cape Town | Chronic stress  due to decentralization  process |
|  | - Supporting the role of middle managers as problem-solving support, embodying respect, empowering others, enabling learning and innovation  - Drawing on social networks and relationships within and outside health system | Kenya and South Africa | District level health system | Chronic stress due  to unstable and  evolving new  governance structures during decentralization |
|  | - Devolution of decision-making to lower system levels | Lebanon and Jordan | National health systems | Increase in refugees during Syrian crisis |
| *Surveillance* | Improving surveillance through emergency operation centers and incident management systems  - Improving messaging (including fearful, educational, and hopeful messaging) | Liberia | District health systems | Ebola |
|  | - Linking health management information systems to broader forecasting trends  - Forecasting longer-term system shocks  Integrating health information with other sectors | Europe and West Africa | National health systems | 2008 financial crisis; climate change disasters; Ebola outbreak; refugee and migration crisis in  Europe |
|  | - New surveillance systems to improve data quality  - Developing remote sensing, early warning systems, and case monitoring systems  - Using surveillance on drug supply and use  - Transferring responsibility to the district level to sustain motivation and improve tracking and control | Sub-Saharan Africa | National health systems | Primarily infectious diseases |
| *Human Resources* | - Providing mental health and psychosocial support to patients and staff  - Allowing staff to work close to home  - Suspending hiring freezes | Palestinian refugees in Syria | UNRWA health system for Palestinian refugees | Syria conflict |
|  | - Discussing human resource issues with health managers and politicians to create solutions  - Task-shifting to support health care workers to do clinical duties  - Engaging nongovernmental organization staff within government facilities  - Establishing new committees with different actors to resolve chronic human resource management stressors | Kenya | Subnational level | Stresses due devolution process |
|  | - Involving health staff in protocol development and community outreach  - Using self-selection as one component of hiring additional staff | USA | State health systems | Domestic Ebola outbreak |
|  | - Development and training for health staff on longer-term planning for what happens after the emergency stage is over | Europe and West Africa | National health systems | 2008 financial crisis; climate change disasters; Ebola outbreak; refugee and migration crisis in  Europe |
|  | - Developing an adequate, trained, and multi-skilled health workforce  - Using performance-based payments  - Continuous on-the-job training | Sub-Saharan Africa | National health systems | Primarily infectious diseases |
| *Communication and collaboration* | - Creating good internal and external communication  - Liaising with different government entities | Palestinian refugees in Syria | UNRWA health system for Palestinian refugees | Syria conflict |
|  | - Coordinating within fora at a central level  - Engaging traditional leaders and establishing presidential engagement  - Having government and Ministry of Health teams co-lead incident management system meetings | Liberia | District health systems | Ebola |
|  | - Including a wide array of service providers from private and public sectors and NGOs  - Establishing multiple sources of funding | Lebanon | National health system | Increase in refugees during Syrian crisis |
|  | - Strengthening communication with the public  - Enhancing public trust in the health system  - Engaging society in tackling public health emergencies | Japan, Iran, South Korea, UK and USA | National health systems | COVID-19 |
| *Preparedness* | - Developing checklists, protocols, and emergency plans  - Including the Ministry of Health as part of command structure in crises | Bangladesh | National health system | Infectious disease outbreaks and natural hazards |
|  | - Integrating response plans with many stakeholder  - Integrating refugees into the national health system | Lebanon | National health system | Increase in refugees during Syrian crisis |
|  | - Developing emergency preparedness plans  - Facilitating access to care through community links  - Increasing advocacy efforts to make sure refugees are acknowledged by host country government | Lebanon and Jordan | National health systems | Increase in refugees during Syrian crisis |
| *Organizational capacity and learning* | - Focusing on organizational capacities such as cognitive capacity, behavioural capacity and contextual capacity | Kenya | Subnational level | Stresses due devolution process |
|  | - Strengthening organizational functioning | Kenya and South Africa | District level health system | Chronic stress due  to unstable and  evolving new  governance structures during decentralization |
|  | - Institutionalising learning  - Defining a learning structure and hub  - Incorporate a learning plan in national health strategies  - Embedding incentives for learning into job descriptions  - Engaging donors to fund technical assistance for learning programmes | Low- and middle-income countries | National health systems |  |
|  | - Investing in structures and processes that promote adaptive capacity of health systems  - Strengthening governance process, leadership practices, organizational culture, and human capital | Global | National health systems |  |
| *Health system strengthening* | - Investing in universal health coverage to protect most vulnerable in crises | Ireland | National health system | 2008 financial crisis |
|  | - Enabling all health facilities to identify, isolate and stabilise infectious diseases until referred  - Developing on-site laboratory capacity | USA | State health systems | Domestic Ebola outbreak |
|  | - Embedding IHR core capabilities in health system strengthening  - Including global health security and IHR as part of medical training worldwide | Japan, Iran, South Korea, UK and USA | National health systems | COVID-19 |
|  | - Develop an action-oriented health system resilience to improve public health capacities within a national context | Global | National health systems | Infectious disease outbreaks, natural hazards |
